# Accuracy and Precision of Silicon Based Impression Media for Quantitative Areal Texture Analysis

**DOI:** 10.1038/srep10800

**Published:** 2015-05-20

**Authors:** Robert H. Goodall, Laurent P. Darras, Mark A. Purnell

**Affiliations:** 1 University of Leicester, Department of Geology, Leicester, UK LE1 7RH

## Abstract

Areal surface texture analysis is becoming widespread across a diverse range of applications, from engineering to ecology. In many studies silicon based impression media are used to replicate surfaces, and the fidelity of replication defines the quality of data collected. However, while different investigators have used different impression media, the fidelity of surface replication has not been subjected to quantitative analysis based on areal texture data. Here we present the results of an analysis of the accuracy and precision with which different silicon based impression media of varying composition and viscosity replicate rough and smooth surfaces. Both accuracy and precision vary greatly between different media. High viscosity media tested show very low accuracy and precision, and most other compounds showed either the same pattern, or low accuracy and high precision, or low precision and high accuracy. Of the media tested, mid viscosity President Jet Regular Body and low viscosity President Jet Light Body (Coltène Whaledent) are the only compounds to show high levels of accuracy and precision on both surface types. Our results show that data acquired from different impression media are not comparable, supporting calls for greater standardisation of methods in areal texture analysis.

Analysis and quantification of natural and manufactured surfaces at micrometric and sub-micrometric scales is becoming widespread. Applications range from engineering^1^ and superconductor technologies in particle accelerators^1^,^2^,^3^,^4^, to archaeology^5^,^6^,^7^, human skin surface topography^8^,^9^, and biomimetics (e.g. antifouling properties of bivalve shells^10^). In particular, quantitative areal surface texture analysis is increasingly applied to analysis of tooth wear as a tool for dietary niche separation (e.g. refs 11, 12, 13, 14, 15, 16, 17, 18, 19, 20, 21).

In many cases, rather than direct analysis of a surface, replicas are used. Often this is for methodological reasons: some samples cannot be transported to the analytical facility, and some are too large to be accommodated by the measuring instruments; some types of surface are prone to movement during measurement (e.g. *in vivo* skin measurements); the properties of some surfaces (e.g. highly transparent or highly reflective) are unsuited for data collection using certain instruments. It is also possible for surface replication to be the solution to certain problems in dentistry caused by the inability of intra-oral dental scanners to collect data at high enough resolution^22^. When replicas are used, data is acquired either from the replica or from a cast made using the replica. Obviously, the quality of data acquired in this way is entirely dependent on the fidelity of surface replication, with significant implications for the accuracy and precision of resulting measurements. Furthermore, if impression media differ in fidelity, this will preclude comparisons between studies based on data acquired using different media.

Clearly, investigations into the precision and accuracy of impression media used are important, but only a few such studies have been conducted^5^,^9^,^10^,^23^,^24^,^25^,^26^,^27^,^28^,^29^,^30^,^31^,^32^, and none have undertaken systematic, statistical comparisons of areal textural parameters acquired from sub-micrometre resolution replicas, produced using a range of impression media with different properties.

Four studies have undertaken qualitative evaluations of impression media used to replicate tooth surfaces for microwear analysis. Two of these^24^,^25^ concluded from visual inspection of SEM images that low viscosity polyvinylsiloxane impression media produced the highest fidelity of replication. Another used similar methods to investigate the fidelity of three moulding compounds of varying viscosity from the President Jet product line (Coltène Whaledent)^30^, concluding that both the low and mid viscosity compounds showed high levels of accuracy. A forth study, investigating accuracy in replicating skin surface textures^9^, used a small number of different impression media, and included no information about the media used. However, none of these studies quantified the variation in resulting surfaces.

Of the remaining studies, very few have directly compared the fidelity with which multiple different compounds replicate the same surface. Most have focussed on a small number of compounds, either to examine the most basic questions of whether a surface can be replicated accurately in the first place^10^,^28^,^31^, or to make recommendations for standard laboratory procedures^23^. Others examined replication at far too coarse a scale (e.g. refs 26,27) to be of use in quantitative areal texture analysis. Analysis of the accuracy of different impression media at replicating sub-micrometre scale surface structure of cuts to bones and tooth surfaces created by tool use in early humans^5^ did not investigate compounds of different viscosity, used only two different impression media, and compared only four parameters (angles within cut marks, derived from 2D profile data). Rodriquez *et al.*^32^ collected 2D profile data to investigate the influence of colour and transparency in a number of impression materials on the accuracy of surface reproduction.

Nilsson and Ohlsson^29^ investigated a range of impression media at the sub-micrometre scale using three dimensional surface texture data, comparing original surfaces to replicas. This study was limited to only three media types, and fidelity was tested only using percentage deviations in surface texture, with no statistical testing of the significance of the differences.

Here we present the results of a quantitative analysis, based on 3D areal texture analysis (see Methods), of the variation in accuracy and precision between seven different silicon based impression media of varying composition and viscosity, investigating their ability to replicate rough and smooth surfaces. For each medium, we present statistical tests of the null hypothesis that areal texture parameters obtained from replicas do not differ from those obtained from the original surface.

Accuracy refers to the degree to which replica surfaces made using different impression media differ from the original surface. We test this through analysis of the number of areal texture parameters that differ significantly when replica and original surfaces are compared. Precision refers to the magnitude of differences for textural parameter values between replicas and original surfaces, and between replicas made using different impression media. As part of this we also test the degree to which differences between original and replica surfaces are systematic rather than random (i.e. do particular impression media consistently increase or decrease parameter values). A moulding compound that produces surfaces with a large number of differences from the original, but all of small magnitude, is inaccurate but relatively precise. An ideal moulding compound would produce surfaces with few significant differences, all of which would be small in magnitude - it would be both accurate and precise. Importantly, we also assess the degree to which imprecision and inaccuracy in replication arising from different moulding compounds are likely to bias the results of analysis. If inaccuracies and imprecision are large in relation to the number and magnitude of differences arising because of variation between different types of original surface under investigation, then their impact on analysis is likely to be significant.

## Results

### Accuracy of Impression Media - ISO 24178-2

**Figure 1:**
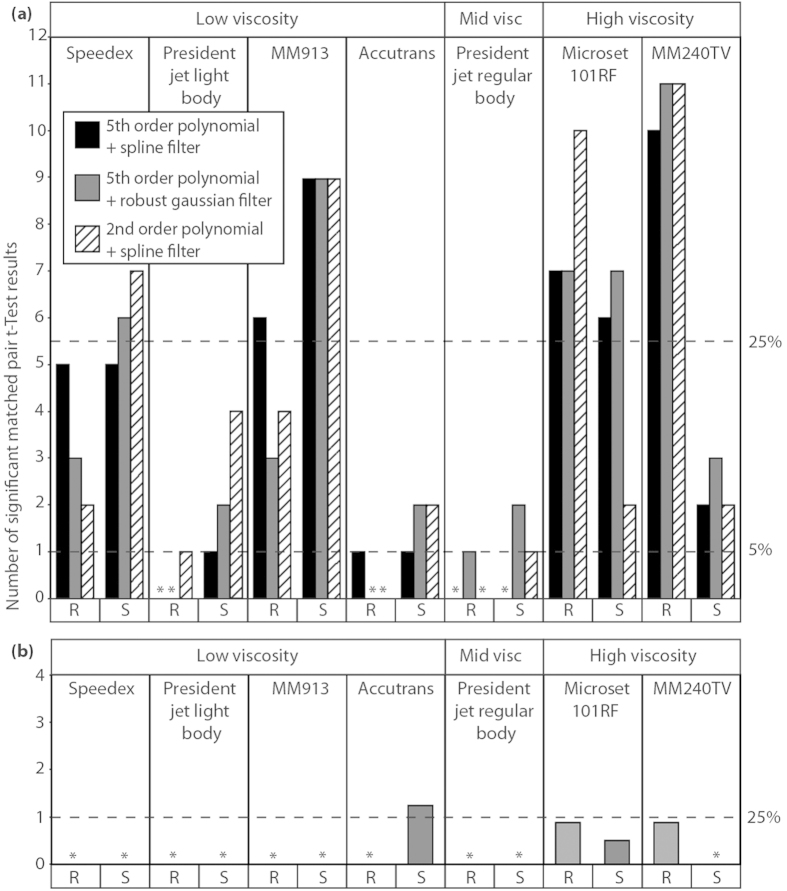
Numbers of significant differences (matched pair t-tests) between impression media and original tooth surfaces. With (**a**) data generated using ISO 25178-2 method and (**b**) data generated using SSFA method. Bars show the number of parameters that differ, (*) represents treatments where no significant results were recorded. For (**a**) data treatments (polynomial/spline/Gaussian filter) reflect different approaches to generation of scale-limited surfaces from which texture parameters are generated. R and S indicate whether data were generated from rough or smooth surfaces, respectively. The dashed line on the Y axis labelled 5% represents the expected number of false positive results per impression medium based on an average of 20.57 tests per impression medium, and α = 0.05. The dashed lines on the Y axes labelled 25% serve to compare numbers of significant results produced using the two different roughness parameterisation methods (ISO & SSFA).

For each impression medium, the null hypothesis of no difference from the original surface was rejected for at least one parameter, but the number of parameters that differed ranged widely: between media, between rough (dentine) and smooth (enameloid) surfaces, and between modes of application (Fig. 1 (a)). To simplify discussion, we report here the average number of significant differences across all three scale limiting settings for each replicating medium, but Fig. 1 (a) shows all differences. For low and mid viscosity media, smooth surfaces exhibited a greater number of significant differences than rough. However the opposite is true for high viscosity media (Microset 101RF and MM240TV).

On the rough surface high viscosity Microset 101RF, and MM240TV produce the greatest number of significant differences, with an average of eight for Microset 101RF, and 10.66 for MM240TV. In MM240TV we also see the largest variation in significant differences between the two surfaces, with an average of 10.66 significant differences on the rough surface, but an average of only 2.33 on the smooth surface. Microset 101RF also displays the highest variability on the smooth surface between results recorded using each of the methods for scale limiting surfaces, varying between two significant differences when using a 2^nd^ order of polynomial and a spline filter, and seven significant differences when using a 5^th^ order of polynomial and a robust Gaussian filter.

The two low viscosity media, MM913 and Speedex, both show high numbers of significant differences across both surface types. They produce smaller numbers of significant differences than high viscosity media in almost all cases (except MM240TV on the smooth surface), but much higher numbers of significant differences than the remaining three low and mid viscosity compounds. The greatest number of significant differences across all impression media on the smooth surface is found in MM913, with an average of nine. The two remaining low viscosity impression media (President Jet Light Body, and Accutrans), along with the mid viscosity President Jet Regular Body, produce the smallest number of significant differences across both surface types with an average of 0.33 significant differences for each of the three compounds on the rough surface, and averages of one significant difference for President Jet Regular Body, 2.33 for President Jet Light Body, and 1.66 for Accutrans on the smooth surface.

**Figure 2:**
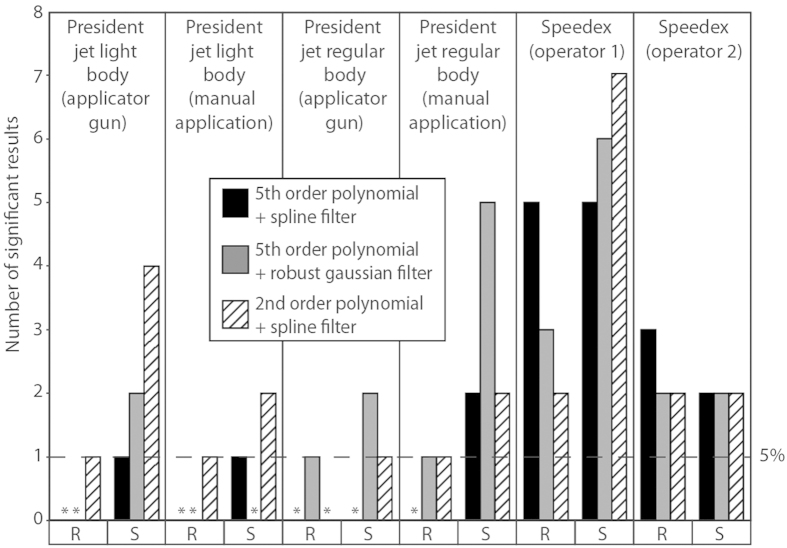
Numbers of significant differences (matched pair t-tests, ISO 25178-2 parameters) between two moulds of the same compound and the original tooth surface. Bars show the number of parameters that differ, (*) represents treatments where no significant results were recorded. Moulds were created using either different operators (Speedex) or application methods (President Jet Light and Regular Body); four quadrants per tooth, broken down by data treatment. R and S indicate whether data were generated from rough or smooth surfaces, respectively. The dashed line on the Y axis (labelled 5%) represents the expected number of false positive results per impression medium based on an average of 20.57 tests per impression medium, and α = 0.05.

Looking at the effect of operator and mode of application (Fig. 2), Speedex shows a great deal of variation in the number of significant differences recorded on both the rough and smooth surfaces, depending on the operator, with moulds produced by operator 1 exhibiting more differences. Comparing applicator gun and manual application, both modes of application of President Jet Light Body to rough surfaces produce few differences. For the smooth surfaces, use of the applicator gun produces a greater number of significant differences than manual application. The converse is true of President Jet regular Body, with manual application to smooth surfaces producing more than twice the number of significant differences compared to using the applicator gun across all scale limiting settings. Manual application to the rough surface also proved less accurate than using the applicator gun, however the difference was only a single significant result in one of the scale limiting settings (2^nd^ order of polynomial with a spline filter).

### Accuracy of Impression Media - Scale Sensitive Fractal Analysis

Comparing impression media to the original surfaces using SSFA parameters yields fewer significant differences (matched pair t-tests) than comparisons using the ISO 25178 method (Fig. 1 (b)). This is partly because SSFA generates fewer parameters. HAsfc is recorded here as a fraction, due to this parameter being calculated across ten different subdivisions (splits) of the sample area.

On the rough surface significant differences were recorded only in the two high viscosity impression media (Microset 101RF & MM240TV), and only in the parameter HAsfc (Surface Heterogeneity; significant differences were recorded in eight of the ten “splits” used to calculate this parameter for each of these impression media).

On the smooth surface there were even fewer significant differences, but they were found in more than one media viscosity level. Again high viscosity Microset 101RF showed significant differences for the parameter HAsfc (in four of the ten “splits” used), however MM240TV recorded no significant differences in any parameter. Significant differences were also found when using low viscosity Accutrans, in the parameters HAsfc (2/10 “splits”), and Asfc (Surface Complexity).

However if we consider the percentage of significant differences, as opposed to the overall number, it may give us a better comparison between the SSFA and ISO 25178 results. In this situation one significant result using SSFA parameters is 25% of the total possible significant differences. If we apply this 25% threshold for significant differences to the ISO 25178 data (5.5 significant differences) we find that it is exceeded by Speedex on the smooth surface, MM913, and Microset 101RF on both surface types, and MM240TV on the rough surface. This is completely different to the pattern seen in the SSFA results, where this threshold is only exceeded by Accutrans on the enameloid surface (1.2 significant differences).

Using SSFA to compare different operators and application methods revealed no difference between application methods.

### Variability in Precision and Accuracy of Impression Media - ISO 24178-2

**Figure 3:**
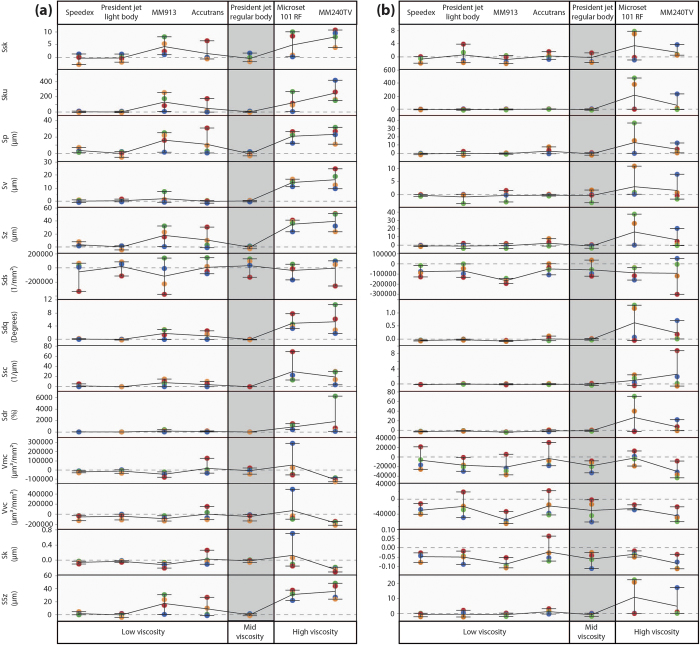
Absolute differences between original surface and each impression medium for the rough surface (**a**), and the smooth surface (**b**), generated using the ISO 25178-2 parameterisation method. Points show the actual differences from the original surface, with zero indicating the same value for replica and original surface. Each quadrant has been given a specific colour (NE = Blue, SE = Green, SW = Red, NW = Orange). Lines connecting points horizontally show mean difference. Whiskers represent the range of the data. For convenience, plot shows only data collected using a 5^th^ order of polynomial and a robust Gaussian filter, and only parameters returning significant differences for at least one impression medium on the rough surface. Other data are included in Supplementary Fig. S1.

We assess precision in terms of the range of deviations in texture parameter values for each impression medium from the original surface values. Rough and smooth surfaces are compared separately; for each parameter and each medium there are four values (one for each quadrant - see Methods), yielding a range of deviations from the original surface (Fig. 3). Because these figures are presented to show differences in accuracy and precision between impression media, plots for the rough and smooth surfaces are given at different scales, and although patterns of variation can be compared, absolute values should be taken into account. For the assessment of precision we have only used the data files that have been scale limited using a 5^th^ order of polynomial and a robust Gaussian filter (as in ref. 20). For clarity, only 13 of the 22 parameters are shown in Fig. 3, all of which represent parameters where at least one significant result was recorded across all impression media on the rough surface. Plots showing data for all remaining parameters are included as Supplementary Fig. S1.

On the rough surface (Fig. 3 (a)) high viscosity media (MM240TV and Microset 101RF) generally show the greatest range of differences from the original surface and thus the lowest precision. Low viscosity media are split into two levels of precision: Accutrans and MM913 show a similar lack of precision to that shown by high viscosity media; President Jet Light Body and Speedex both show very high levels of precision, with differences clustered much more closely. Finally President Jet Regular Body shows a similarly high level of precision to Speedex and President Jet Light Body, with very little to clearly differentiate the precision of the three compounds. The precision of each impression medium appears to mirror its accuracy on the rough surface, with compounds showing low accuracy also generally showing low precision and vice versa. However, there are two notable exceptions to this pattern, Speedex, which shows high precision, but low accuracy, and Accutrans, which shows high accuracy, but low precision. Microset 101RF shows a much higher level of precision than is typical for this medium in one or two parameters.

On the smooth surface (Fig. 3 (b)) the pattern of precision is slightly different. The two President Jet compounds and Speedex show a similar high level of precision to that seen on the rough surface. The two high viscosity media (Microset 101RF and MM240TV) again show low levels of precision. However Accutrans and MM913 show much higher levels of precision on the smooth surface, similar to that seen in the two President Jet compounds and Speedex. In most cases, deviations from the original surface values on the smooth surface are smaller in scale than on the rough. However, this is not the case for height parameters, where differences on the smooth surface are similar, and sometimes larger, than those on the rough surface. There appears to be a homogenisation of the precision between the four low viscosity and the one mid viscosity impression media on the smooth surface, making it much harder to determine within these compounds which has the highest precision. For the volume parameters Vmc and Vvc, and the material ratio parameter Sk, all media show a similar level of precision.

On both the rough and smooth surfaces there is a degree of directionality in the error produced by the four least precise media (MM240TV, Microset 101RF, Accutrans and MM913). This is because, for certain parameters, the differences from the original surface are mostly either positive or negative. This implies there is a consistent bias (e.g. a constantly positive bias for parameter Sp would indicate elevated peak heights). However, any bias is not systematic as the order of each quadrant’s difference from the original surface is never repeated (i.e. NW quadrant does not consistently have the largest error across all compounds and parameters) (Fig. 3). For the results of any parameter to be considered to have positive directionality of error at least three of these four media must show mostly positive differences from the original surface (more than 50% of quadrants in more than 50% of media), and vice versa for negative directionality of error. Both rough and smooth surfaces show an equal degree of directionality, with 12 parameters showing either positive or negative directionality of error on each surface type. There are ten parameters on each of the surface types, in which there is no obvious directionality in differences from the original surface.

There is a small number of parameters where the directionality of error is consistent across both surface types. On both the rough and smooth surface there is positive directionality in the Hybrid Parameter Sdr, the Material Ratio Parameter Svk and the Feature Parameter S5z. And there is consistent negatively directionality across both surface types for the Spatial Parameter Str, and the Volume Parameter Vvc.

However, most parameters only show directionality of error on one of the two surface types. Positive directionality is also seen on the rough surface in the Height Parameters Ssk, Sku, Sp, Sv, and Sz, and the Hybrid Parameters Sdq and Ssc, and on the smooth surface in the Volume Parameter Vvv. Negative directionality of error is also seen on the smooth surface in the Hybrid Parameter Sds, the Volumetric Parameters Vmp, and Vmc, and the Material Ratio Parameters Sk, Smr1, and Smr2.

### Variability in Precision and Accuracy of Impression Media - Scale Sensitive Fractal Analysis

**Figure 4:**
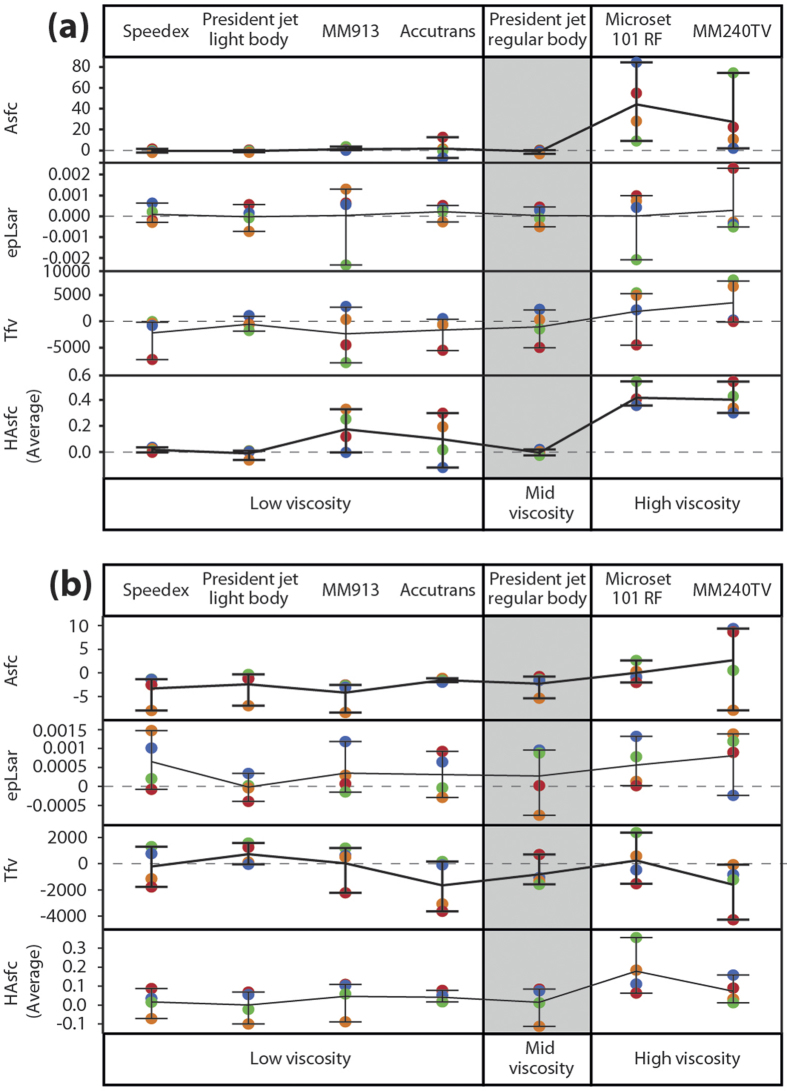
Absolute differences between original surface and each impression medium for the rough surface (**a**), and the smooth surface (**b**), generated using the SSFA parameterisation method. Points show the actual differences from the original surface, with zero indicating the same value for replica and original surface. Each quadrant has been given a specific colour (NE = Blue, SE = Green, SW = Red, NW = Orange). Lines connecting points horizontally show mean difference. Whiskers represent the range of the data.

The precision of impression media when using SSFA parameters was assessed in the same way as with ISO parameters above (Fig. 4). On both surface types there appear to be different patterns of precision depending on the medium and parameter in question. In some media this pattern is similar across both surface types, however in others the two surface types show very different patterns of precision. This is markedly different to the ISO parameter data, where the patterns were similar across most parameters and across the two surface types. Therefore it appears that in this case differences between media are less systematic when using the SSFA parameterisation method than those detected using the ISO-based analysis.

On the rough surface (Fig. 4 (a)), Speedex, President Jet Light Body and President Jet Regular Body all show very high levels of precision for parameter Asfc (surface complexity), and HAsfc (heterogeneity), but much lower precision for epLsar (anisotropy) and Tfv (textural fill volume), giving them a similar level of precision to Accutrans for these two parameters. Accutrans is less precise than Speedex and President Jet media for other parameters, but in all but one case precision is better than the remaining three media (the exception is HAsfc, with Accutrans showing the lowest levels of precision of any media on the rough surface).

Low viscosity MM913, and the two high viscosity media (Microset 101RF and MM240TV), all generally show very low levels of accuracy on the rough surface. However MM913 shows much higher levels of precision for the parameter Asfc, similar to the precision seen in the President Jet media and Speedex.

On the smooth surface (Fig. 4 (b)) all impression media show low levels of precision for parameters Asfc, epLsar, Tfv, and HAsfc, without much to separate them. Except in the case of Accutrans, where higher levels of precision can be seen for the parameters Asfc and HAsfc than for the other media.

Although the pattern of precision for the rough surface is similar to that seen when using the ISO parameterisation method, the pattern on the smooth surface is different. On both surface types there is also very little directionality of error evident when using the SSFA parameterisation method.

### Magnitude of Differences Between Surfaces: Replicas Compared to Different Diets

Comparisons of precision and accuracy provide a good test of the fidelity of each of the impression media, but they do not address the question of whether the magnitude of differences that result from using different media would produce erroneous results in a comparative statistical analysis. This kind of analysis is routinely used to investigate dietary differences between species or ecotypes of vertebrates based on differences in 3D microtexture of tooth surfaces. Here we compare the magnitude of the differences in parameter values between different media with the differences obtained from comparing surface textures of teeth from two wild populations of *Archosargus probatocephalus* (Sheepshead Seabream) which exhibit different tooth surface microtextures as a result of dietary differences (this is the same species as that from which our other surface data were acquired). Both populations were collected in Florida (USA) and although they can be considered as dietary generalists with considerable overlap in diet, one population, from Indian River lagoon, is more herbivorous, while the other, from Port Canaveral lagoon, consumes and crushes more hard-shelled prey, such as bivalves^33^.

**Figure 5:**
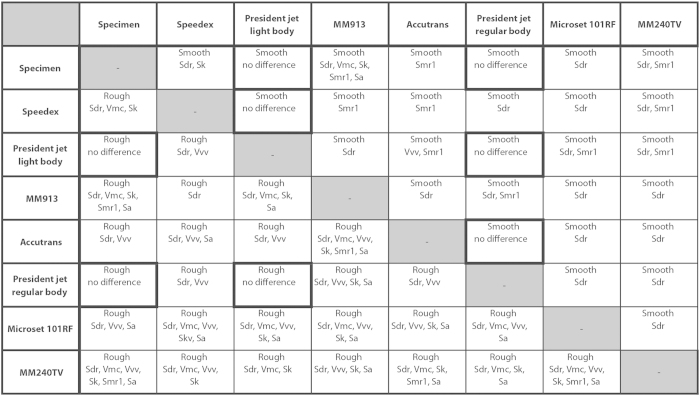
Magnitude of differences in texture parameters between impression media compared to the magnitude of differences between dietary ecotypes of *Archosargus probatocephalus*. Only seven ISO 25178-2 parameters (Sdq, Sdr, Vmc, Vvv, Sk, Smr1, and Sa) were used, as these were the only ones to differ significantly between the two *Archosargus probatocephalus* dietary populations^33^. The boxes show those parameters where differences between replica surfaces and the original tooth surfaces exceed those reflecting dietary differences; all possible pairwise comparisons between impression media and the original tooth surfaces were assessed. Whether a parameter value exceeds the dietary difference is calculated by comparing the median value of differences between surfaces (e.g. between the original specimen and Speedex) with the difference between the median value of each the dietary ecotypes. Information towards the lower left shows results for the rough surface, information toward the upper right for the smooth surface. The parameter Sdq is not shown because it exceeds the value for the dietary difference in 27 of 28 comparisons on both surfaces and thus tells us nothing about the relative potential of different impression media to introduce bias into the results of dietary analysis. Highlighted cells represent comparisons where no difference equalled or exceeded that expected from two dietary populations (not including Sdq).

In the dietary analysis, seven ISO 25178-2 parameters (Sdq, Sdr, Vmc, Vvv, Sk, Smr1, and Sa) differed significantly between populations^33^. Figure 5 shows the results of comparing the magnitude of differences between each impression medium, and the original surface with the magnitude of differences between dietary groups for these seven parameters. The parameters listed in each box are those that exhibit a difference between impression media of greater magnitude than would be expected between the different dietary groups.

We find that only two impression media return no differences of greater magnitude than would be expected between dietary groups across both surface types: President Jet Regular Body and President Jet Light Body. All other comparisons between impression media and against the original surface return differences of greater magnitude than would be expected between two dietary populations for at least one parameter, but often more. The number of parameters showing greater magnitude than expected between dietary groups is much smaller on the smooth surface than on the rough surface.

When comparing the magnitude of inter-individual differences within each dietary population to the differences between impression media on the smooth surface we see an almost identical pattern (Supplementary Fig. S2) to that shown above.

## Discussion

It is clear that different impression media differ significantly in their ability to accurately and precisely replicate surfaces. Accuracy and precision vary between smooth and rough surfaces, between compounds with different levels of viscosity, and between compounds of similar viscosity.

When using the ISO parameterisation method, high viscosity media (Microset101RF and MM240TV) show the lowest accuracy and precision when replicating a rough surface, at the scale used here, although there is some variation between different data treatments. Many more significant differences are found than low or medium viscosity media in almost all cases, and the magnitude and range of differences from the original surface is much higher than most other media. However MM240TV shows relatively high accuracy on the smooth surface. Comparing profiles across equivalent surfaces produced using different impression media suggests that the higher viscosity of these compounds limits their ability flow into, and thus replicate, the smallest scale features of the surface topology.

Low viscosity media generally replicate a surface more accurately and precisely than high viscosity media, but this is an oversimplification. The number of significant differences and the range of differences from the original surface vary between low viscosity media and between data treatments and the data suggest that all low viscosity compounds are less accurate when replicating a smoother surface at the sub-micrometre scale. On the rough surface President Jet Light Body and Accutrans appear to be the most accurate low viscosity media, showing very few significant differences across all data treatments. However, although President Jet Light Body shows a high level of precision, especially on the rough surface, Accutrans shows much lower precision, similar to the high viscosity media. On the smooth surface both compounds show high levels of precision, with very little difference in precision between these two compounds. Speedex and MM913 appear to be much less accurate on both the rough and smooth surface and show a number of consistent significant differences, across data treatments. On the rough surface, MM913 shows a consistently low level of precision across all parameters, however Speedex is much more precise. On the smooth surface Speedex and MM913 showed a relatively high level of precision in most parameters. The accuracy of Speedex varied greatly depending on the operator applying the impression medium; both operators were experienced in the use of this compound, and it is unlikely that variation was caused by operator competence; our results therefore suggest there may be issues with using this compound, probably linked to the need to manually measure out and mix imprecise volumes of medium and activator before use. The same might be true of other manually mixed compounds.

President Jet Regular Body, the only mid viscosity impression medium studied, showed the lowest number of significant differences across both surface types and between all data treatments. For President Jet Regular body, given that our multiple comparisons would lead us to expect about one false positive result in every 20 tests, and the fact that there is very little consistency between different data treatments, we would suggest that for the significant differences found when comparing this compound to the original teeth we cannot reject the hypothesis that these are mostly type I errors resulting from multiple comparisons. Also, on the rough surface President Jet Regular Body is one of three compounds showing the highest level of precision, (and shows among the highest levels of precision for most parameters on the smooth surface). It is also one of only two compounds not to show any differences from the original surface of a magnitude greater than that seen between different dietary groups. Manual application of President Jet Regular Body produces higher numbers of significant differences on the smooth surface, possibly because the medium is too viscous to be applied consistently in this way.

When looking at the four media with lowest precision, the directionality of error can tell us something about how the replicated surface differs from the original. Focusing on the parameters that show consistent directionality of error across both surface types, MM913, Accutrans, Microset 101RF, and MM240TV generally over replicate the developed interfacial area ratio (Sdr), the mean depth of valleys below the core material (Svk), and the average value of the five highest and lowest peaks (S5z), and under replicate the surface texture aspect ratio (Str), and the surface core void volume (Vvc) of both smooth and rough surfaces. It is also clear that these compounds generally over replicate most height parameters on the rough surface, and under replicate both peak and valley material portions on the smooth surface. There is also under replication of core void volumes on the rough surface, and over replication of valley void volumes on the smooth surface.

Finally, it appears that there are marked differences between the two surface roughness parameterisation methods currently used in the study of vertebrate diet. The Scale Sensitive Fractal Analysis method produces far fewer significant differences than the ISO 25178-2 method, even when the large difference in numbers of parameters between the two methods is accounted for. The SSFA method also shows no clear pattern on the smooth surface when it comes to understanding the precision of different media. It is unclear whether the differences we see between these methods arise because SSFA is less sensitive, or because the ISO method is exaggerating differences in the surfaces. Further work is needed to understand this.

Given their inaccuracy and imprecision, high viscosity compounds should not be used to replicate surfaces when quantifying 3D areal textures at sub-micrometre scales. Our results also suggest that there are problems with at least two of the low viscosity compounds tested - Speedex and MM913 - on both rough and smooth surfaces. MM913 is slightly less accurate than Speedex on both surfaces, and much less precise on the rough surface, and Speedex shows some potential for operator error to play a part in results. President Jet Light Body may have an issue when studying smooth surfaces, however the level of inaccuracy is very variable and, alongside the generally high precision seen for this compound, it should not be completely discounted. President Jet Light Body does however have a short cure time, which can cause problems when moulding large surfaces.

Low viscosity Accutrans and mid viscosity President Jet Regular Body show the highest accuracy, producing the lowest number of significant differences across both surface types. However Accutrans shows a low level of precision, especially on the rough surface. The only caveat to using President Jet Regular Body is that manual application will produce less accurate and less precise data, and our results support the use of an applicator gun. On smooth surfaces, President Jet Regular Body shows higher accuracy than Accutrans, and on rough surfaces its shows higher levels of precision.

President Jet Light and Regular Body are also the only two compounds that do not show differences when compared to original surfaces, or to each other, that are greater in magnitude than those found between dietary groups. In the context of dietary analysis based on tooth microwear, we would therefore not recommend that surfaces obtained from impression media other than President Jet Light or Regular body are compared either with each other or with original surfaces. Such comparisons are likely to produce erroneous differences reflecting replication, not ecology.

For most impression media, our results lead to rejection of our null hypothesis that areal texture parameters obtained from replicas do not differ from those obtained from the original surface. Impression media vary in their ability to accurately and precisely reproduce a given surface, with most producing statistically significant differences, and high deviations from true values for areal texture parameters derived from original surfaces, even when false positive results are taken into account. Of the media tested here, President Jet Regular Body produced the most accurate and precise surface replicas.

## Methods

### Materials

The lower right jaw (dentary) of an adult specimen of *Archosargus probatocephalus* (Perciformes: Sparidae) was dissected and mounted on a temporary base to facilitate manipulation. Two worn teeth with obvious variation in surface texture were selected from among the molariform teeth of the jaw: one exhibiting little wear, with a relatively smooth, enameloid surface; the other, more worn, with a relatively rough surface of exposed dentine (the enameloid having been worn away). A needle was used to scratch two intersecting perpendicular lines across the centre of each tooth surface, dividing it into quadrants. Within each quadrant a relocatable 100 × 145 μm area was identified, based on recognisable surface features, so that data could be collected from the same location on the replicated surfaces (Supplementary Fig. S3; areas designated NE, SE, SW, NW). Before the moulds used in this study were collected, tooth surfaces were cleaned by applying a random light body impression medium to the surfaces, which was then discarded.

Seven impression media were selected, representing different viscosity levels (Table 1). Four are polyvinylsiloxane compounds, two room temperature vulcanising (RTV) rubber compounds, and one heat accelerated RTV compound. Moulds were taken using each of the different media in a random order. Some media allow use of an applicator gun, which standardizes the mixing of two-components by extruding them through a helical nozzle; others required the body and activator components to be mixed and applied manually.

For each medium we tested accuracy and precision of replication, and for three media we also tested the effect of how they were applied (manual versus applicator gun, and application by different operators). The latter test was based on moulds taken using three different impression media, representing the compounds currently used in dietary microwear analysis: two moulds of manually mixed Speedex, each made by a different operator, to test for effects of variability between operators; two moulds of President Jet Light Body, one applied to the surface using the applicator gun, the other applied manually; two moulds of President Jet Regular Body, one applied to the surface using the applicator gun, the other applied manually. Manual versus applicator comparison was not possible with Speedex, because an applicator version is not available.

Epoxy casts were produced from each mould using EpoTek 320LV. In many studies, particularly of tooth microwear, transparent/translucent epoxy casting material is used, but in order to optimise data acquisition (using focus variation microscopy; see below) we used the black pigmented EpoTek 320LV, which in other respects has similar properties to the commonly used transparent EpoTek 301. After all moulds were taken, data were acquired from the original tooth surfaces (gold coated, using an Emitech K500X sputter coater, for three minutes to optimise data acquisition). Throughout the text, each cast is referred to by the name of the impression media from which it was created.

### Data Acquisition

3D surface texture data were collected using focus variation microscopy (Alicona Infinite Focus Microscope, model IFM G4c, software version: 2.1.2). Data capture followed the methods of previous studies^13^,^20^,^21^ (x100 objective, field of view of 145 × 110 μm, vertical resolution set to 0.02 μm, lateral optical resolution equivalent to 0.35–0.4 μm). Data were captured from exactly the same fields of view across all replicas, and from the original tooth surfaces, so that for each quadrant (NE, SE, SW, and NW), there is an identical sample area for the original surface and each replica (see Supplementary Fig. S3 for examples of 3D surface data).

The resulting data files were investigated using two different approaches to surface texture analysis: one based on ISO 25178-2^1,34^, the other using Scale Sensitive Fractal Analysis. In the first, data files were levelled using all points levelling (fit to a least squares plane via rotation around all three axes) to remove any variation in the 3D surface arising from manual horizontal positioning of the sample. Files were then transferred to SurfStand software (Version: 5.0.0) for further processing. Errors in data collection (e.g. data spikes) were manually deleted and replaced with a mean surface value point. Surface roughness was quantified using ISO 25178-2 texture parameters (Table 2) which requires generation of scale-limited surfaces^34^ (for detailed parameter descriptions see refs 17,20). Scale limited surfaces were generated through application of a robust polynomial (which finds and removes the Least Squares polynomial surface for the levelled data) combined with either a spline or a robust Gaussian wavelength filter (to remove long wavelength features of the tooth surface; gross tooth form). Three different settings were used, producing three complete datasets of eight samples: a 2^nd^ order polynomial with a spline filter, a 5^th^ order polynomial with a spline filter, and a 5^th^ order polynomial with a robust Gaussian filter, all with the wavelength cut-off for the filter set to 0.025 mm. This allowed us to account for differences in the process of generating scale-limited surfaces causing variation in assessments of accuracy and precision. Two of the three settings also correspond to previous work carried out on dietary analysis based on ISO texture parameters^20^,^33^.

Scale Sensitive Fractal Analysis (SSFA)^16^,^17^ was carried out using the programs ToothFrax and SFrax (Surfract, www.surfract.com). SSFA does not require surfaces to be scale limited, and quantifies five aspects of surface roughness (Table 3). Settings for all parameters followed those used in previous work^17^, including the use of scale-sensitive “auto splits” to record Surface Heterogeneity (HAsfc), separating individual scanned sections into increasingly reduced sub-regions (we calculated HAsfc across ten different subdivisions). As a small deviation from the published method we used a single data file location for each sampled surface, rather than four adjoining locations normally used. This was necessary in order for us to directly compare the same locations from which ISO parameter data were calculated. Also, rather than a setting of 1.8 μm^17^, we used a 3.5 μm scale of observation to calculate the parameter epLsar^35^ (this value being based on the lateral resolution of the microscope being used).

### Statistical Analysis

Statistical hypothesis testing was carried out using JMP (Version 10.0.0). Data acquired from rough and smooth surfaces were analysed separately. Data sets were tested for normality (Shapiro Wilks W test; by parameter and impression medium); the majority of data were normally distributed so parametric statistical tests were appropriate. Log_10_ data were used for parameters where this produced a greater number of normally distributed media. For each parameter either original data or log_10_ data were used across all media, never a combination of the two. The ISO 25178-2 parameter Sal (Auto-Correlation Length), and the SSFA parameter Smc (Scale of Maximum Complexity) were found rarely to be normally distributed in any impression medium and were excluded from further analysis.

Because data were collected from exactly the same eight locations on the two teeth and each set of replicas, our replica datasets can be considered as ‘treatments’ of the original surfaces. Consequently we tested for differences using matched pair t-tests, so that rather than treating the data from a replica as a general sample population, the same quadrants are compared (e.g. comparing the Microset replica with the original surface, Microset data for the NE quadrant are compared with original data for the NE quadrant, Microset SE compared with original SE etc.)

Although we conducted multiple comparisons, a sequential Bonferroni correction^36^ was not applied, because knowing when to use this method is difficult and in most cases subjective^37^; when used on test numbers as large as ours, the correction has been shown to produce more type II error (false negatives) than the type I error (false positives) it removes^38^,^39^. Choosing not to use a Bonferroni correction will bias our results towards incorrectly rejecting the null hypothesis of no difference between moulding compounds (i.e. it will increase the likelihood of type I errors), and this is taken into account when drawing our conclusions (e.g. given that an average of 20.57 tests were performed for each impression medium using the ISO 25178-2 data, we might expect, at α = 0.05, one false positive for each medium).

## Author Contributions

M.A.P conceived and designed research programme. R.H.G and M.A.P wrote the main manuscript text. R.H.G and L.P.D generated data. M.A.P and R.H.G analysed and interpreted results. R.H.G and M.A.P prepared all figures and tables. All authors reviewed the manuscript.

## Additional Information

**How to cite this article**: Goodall, R. H. *et al.* Accuracy and Precision of Silicon Based Impression Media for Quantitative Areal Texture Analysis. *Sci. Rep.* **5**, 10800; doi: 10.1038/srep10800 (2015).

## Supplementary Material

Supplementary Information

## Figures and Tables

**Table 1:** Details of all seven silicon based impression media used in this study.

**Impression Media**	**Application**	**Viscosity Level**	**Manufacturer**	**Colour**
Speedex Light Body	Manual	Low	Coltène-Whaledent	Blue
President Jet Light Body	Applicator Gun	Low	Coltène-Whaledent	Green
MM913	Manual	Low	ACC Silicones	Transparent
Accutrans	Applicator Gun	Low	Coltène-Whaledent	Brown
President Jet Regular Body	Applicator Gun	Medium	Coltène-Whaledent	Blue
Microset 101RF	Applicator Gun	High	Microset Products Ltd	Black
MM240TV	Manual	High	ACC Silicones	Light Blue

Speedex, President Jet Light and Regular Body, and Accutrans are polyvinylsiloxane compounds. MM913 and MM240TV are room temperature vulcanising (RTV) rubber compounds, and Microset 101RF is a heat accelerated RTV rubber compound.

**Table 2:** ISO 25178-2 parameters used, including brief descriptions.

**Table 3:** Scale Sensitive Fractal Analysis (SSFA) parameters used, including brief descriptions (after refs.^16^,^17^)

**Parameter Name**	**Acronym**	**Description**
Area Scale Fractal Complexity	Asfc	A measure of the complexity of a surface. Area-scale fractal complexity is a measure of change in roughness with scale. The faster a measured surface area increases with resolution, the more complex the surface.
Exact Proportion Length Scale Anisotropy of Relief	epLsar	A measure of the anisotropy of a surface. Anisotropy is characterized as variation in lengths of transect lines measured at a given scale (we use 3.5 μm) with orientations sampled at 5° intervals across a surface. An anisotropic surface will have shorter transects in the direction of the surface pattern than perpendicular to it (e.g. a transect that cross-cuts parallel scratches must trace the peaks and valleys of each individual feature)
Scale of Maximum Complexity	Smc	The parameter represents the full scale range over which Asfc is calculated. High Smc values should correspond to more complex coarse features
Textural Fill Volume	Tfv	The total volume filled (Tfv) is a function of two components: 1) the shape of the surface, and 2) the texture of the surface. A more concave or convex surface will have a larger total fill volume than a planar surface even if both surfaces have an identical texture
Heterogeneity of Area Scale Fractal Complexity	HAsfc	Variation of Asfc across a surface (across multiple, equal subdivisions of a surface). High HAsfc values are observed for surfaces that vary in complexity across a facet

Smc was excluded from statistical analyses as it was rarely normally distributed and almost always returned the same value for each surface. For parameter details and information on methods of calculation see ref. 17.
