# Supplementary material for: Accuracy and Precision of Silicon Based Impression Media for Quantitative Areal Texture Analysis
Source: Sci Rep. 2015 May 20;5:10800. doi: 10.1038/srep10800 (PMC4438484; doi:10.1038/srep10800)
Supplement: Supplementary Information [file srep10800-s1.pdf]

ACCURACY AND PRECISION OF SILICON BASED IMPRESSION MEDIA FOR QUANTITATIVE AREAL SURFACE TEXTURE ANALYSIS

Goodall, Robert. H.[1], Darras, Laurent. P.[1], *Purnell, Mark. A.[1]
[1]University of Leicester, Department of Geology, Leicester, UK LE1 7RH
* Correspondence to mark.purnell@le.ac.uk

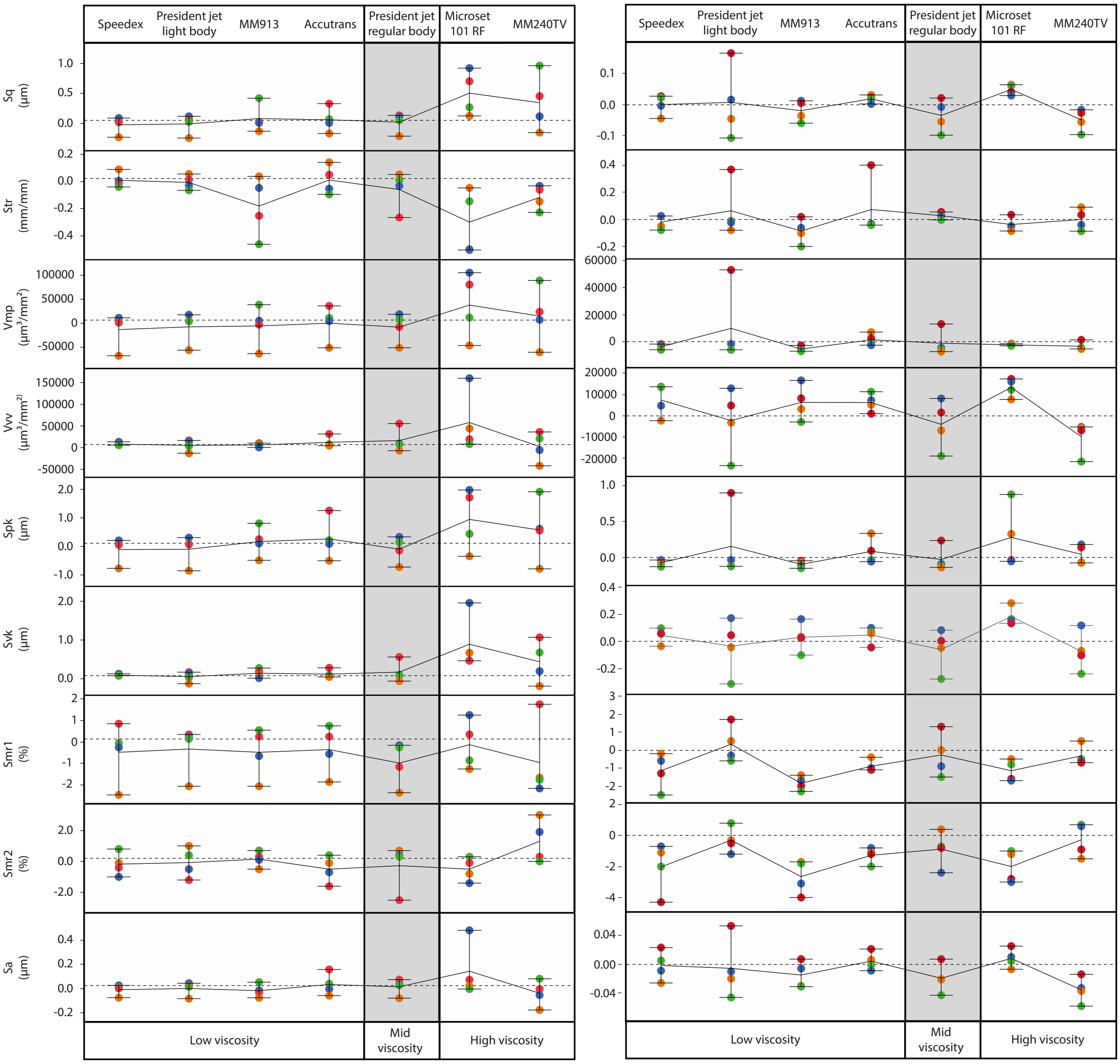

Supplementary Figure S1. Absolute differences between original surface and each impression medium for the rough surface (a), and the smooth surface (b), generated using the ISO 25178-2 parameterisation method. Points show the actual differences from the original surface, with zero indicating the same value for replica and original surface. Each quadrant has been given a specific colour (NE=Blue, SE=Green, SW=Red, NW=Orange). Lines connecting points horizontally show mean difference. Whiskers represent the range of the data. For convenience, plot shows only data collected using a 5th order of polynomial and a robust Gaussian filter, and only parameters not returning significant differences for at least one impression medium on the rough surface.

	Specimen	Speedex	President jet light body	MM913	Accutrans	President jet regular body	Microset 101RF	MM240TV
**Specimen**	-	PC population Sdr, Sk, Sa	PC population no difference	PC population Sdr, Vmc, Sk, Smr1, Sa	PC population Vvv, Smr1	PC population no difference	PC population Sdr	PC population Sdr, Sk, Smr1, Sa
**Speedex**	IR population Sdr	-	PC population Sdr	PC population Smr1	PC population Vvv, Smr1	PC population Sdr	PC population Sdr	PC population Sdr
**President jet light body**	IR population no difference	IR population no difference	-	PC population Sdr, Smr1	PC population Vvv, Smr1	PC population no difference	PC population Sdr, Smr1	PC population Sdr, Smr1
**MM913**	IR population Sdr, Smr1	IR population Smr1	IR population Sdr, Smr1	-	PC population Sdr, Vvv, Sk, Sa	PC population Sdr, Smr1	PC population Sdr, Sa	PC population Sdr
**Accutrans**	IR population Smr1	IR population Smr1	IR population Smr1	IR population Sdr	-	PC population no difference	PC population Sdr	PC population Sdr
**President jet regular body**	IR population no difference	IR population no difference	IR population no difference	IR population Sdr, Smr1	IR population no difference	-	PC population Sdr	PC population Sdr
**Microset 101RF**	IR population Sdr	IR population Sdr	IR population Sdr, Smr1	IR population Sdr	IR population Sdr	IR population Sdr	-	PC population Sdr
**MM240TV**	IR population Sdr, Smr1	IR population Sdr	IR population Sdr, Smr1	IR population Sdr	IR population Sdr	IR population Sdr	IR population Sdr	-

Supplementary Figure S2. Magnitude of differences in texture parameters between impression media compared to the magnitude of differences between individuals in two populations (dietary ecotypes) of Archosargus probatocephalus (compared to smooth tooth surface). The boxes show those parameters where differences between replica surfaces and the original tooth surfaces exceed those between individuals in a population; all possible pairwise comparisons between impression media and the original tooth surfaces were assessed. Both fish populations are from Florida, USA: the Indian River lagoon population is more herbivorous, while the Port Canaveral lagoon population consumes and crushes more hard-shelled prey, such as bivalves. Only seven ISO 25178-2 parameters (Sdq, Sdr, Vmc, Vvv, Sk, Smr1, and Sa) were compared, as these were the only ones to differ significantly between the two A. probatocephalus populations 31. Whether a parameter value exceeds the dietary difference is calculated by comparing the median value of differences between surfaces (e.g. between the original specimen and Speedex) with the median value of differences between individuals in each population. Information towards the lower left shows results for the comparisons with the Indian River population, information toward the upper right for the Port Canaveral population. The parameter Sdq is not shown because it exceeds the value for the dietary difference in almost all comparisons and thus tells us nothing about the relative potential of different impression media to introduce bias. Highlighted cells represent comparisons where no difference equalled or exceeded that expected from within a wild population (not including Sdq).

Rough Tooth	Summary of Results	
Impression Medium	Accuracy	Precision
Speedex	x	✓
President Jet Light Body	✓	✓
MM913	x	x
Accutrans	✓	x
President Jet Regular Body	✓	✓
Microset 101RF	x	x
MM240TV	x	x

Smooth Tooth	Summary of Results	
Impression Medium	Accuracy	Precision
Speedex	x	✓
President Jet Light Body	x	✓
MM913	x	✓
Accutrans	x	✓
President Jet Regular Body	✓	✓
Microset 101RF	x	x
MM240TV	x	x

Supplementary Figure S3. Summary of overall Accuracy and Precision for each impression medium, separated across rough and smooth tooth surfaces. For convenience all treatments of the data are summarised as a single result. Impression media showing high Accuracy (one or fewer significant matched pair T-test results across all treatments of the data) or high Precision (a small range of absolute differences between the original surface and each impression medium) are marked with a (✓). Impression media showing low Accuracy (more than one significant matched pair T-test results across all treatments of the data) or low Precision (a medium to high range of absolute differences between the original surface and each impression mediumare marked with an (x). Results are highlighted green for instances where both Accuracy and Precision are shown to be high in a given impression medium.

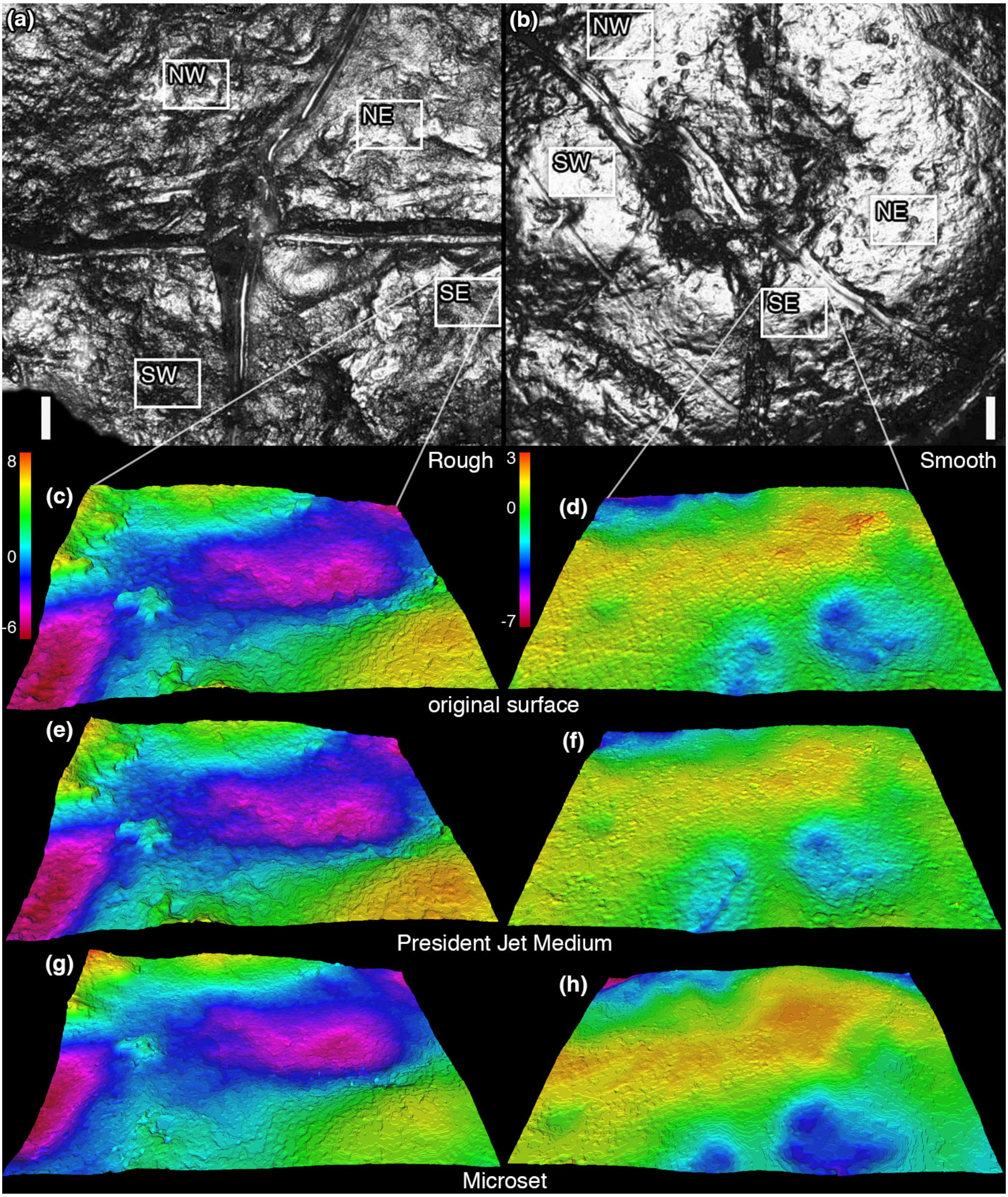

Supplementary Figure S4. Sample locations of four quadrants from the rough (a) and smooth (b) tooth surfaces (optical images). (c) - (h), digital elevation models of levelled surface data from original surface and examples of replicas made using different impresion media for SE quadrant, for rough (c, e, g) and smooth (d, f, h) surfaces. (c) and (d) original surfaces; (e) and (f) replicas, President Jet medium body impression medium; (g) and (h) replicas, Microset impression medium. Scale bars in (a) and (b), 100μm. Digital elevation models all 110 x 145 μm. Vertical scales in μm.
